# Adhesive Closed-loop Small Bowel Obstruction

**DOI:** 10.5811/cpcem.2017.10.35927

**Published:** 2018-01-09

**Authors:** Matthew K. Edwards, Christopher S. Kuppler, Chasen A. Croft, Hannah M. Eason-Bates

**Affiliations:** University of Florida Health, Department of Emergency Medicine, Gainesville, Florida

## Abstract

Complete small bowel obstruction (SBO) is a common surgical emergency often resulting from adhesive bands that form following iatrogenic peritoneal injury. Rarely, adhesive SBO may arise without previous intra-abdominal surgery through other modes of peritoneal trauma. We present the case of a male evaluated in the emergency department for a closed-loop small bowel obstruction due to an adhesive band that likely formed after blunt abdominal trauma over two decades earlier. We review the epidemiology, pathophysiology, and treatment options for similar cases of adhesive SBO.

## INTRODUCTION

Small bowel obstruction (SBO) is a common clinical entity in emergency medicine and globally remains an important source of morbidity and mortality. Approximately 65–75% of SBOs are due to peritoneal adhesions, aberrant fibrous bands within the abdominal cavity that constrict the intestine and disrupt its luminal flow.^1^–^2^ Peritoneal adhesions represent a considerable burden to patients and healthcare systems, annually causing more than 350,000 hospital admissions leading to over 960,000 days of inpatient care and $2.3 billion in medical expenditures in the United States.^3^ Injury sustained during intra-abdominal surgery accounts for the majority of adhesions causing SBO, with the remainder often attributed to peritonitis or congenital formation.^4^ Rarely, however, adhesive SBO may occur without prior abdominal surgery through other modes of peritoneal injury. We examine the unusual case of a patient with a closed-loop SBO secondary to an omental band adhesion likely associated with a remote history of blunt abdominal trauma.

## CASE REPORT

A 37-year-old male was evaluated in our emergency department for a 16-hour history of constant, cramping epigastric abdominal pain and nausea. His past medical history was significant for a pelvic fracture, suffered during a motor vehicle collision (MVC) more than 20 years earlier, which required open reduction and internal fixation at the pubic symphysis. The patient reported no previous abdominal surgeries, recent abdominal injury, or history of acute or chronic intra-abdominal inflammation other than that likely associated with his remote abdominal trauma. He was otherwise in good health and did not use any regular medications.

The patient’s vital signs were stable upon presentation, including heart rate of 75 beats per minute, oxygen saturation of 95%, respiratory rate of 14 breaths per minute, and temperature of 36.4 degrees Celsius. His physical examination was notable for dry mucous membranes, active bowel sounds, and tenderness in the epigastrium without abdominal guarding, distention, or palpable mass. During this evaluation, the patient experienced paroxysms of worsened abdominal pain that at times caused him to retch. The quality and location of his pain, along with his arrival to the hospital near midnight, raised our suspicion for an intestinal obstruction. The laboratory testing showed an elevated white blood count (12.1×10^9^/liter, normal 4.0–10.0×10^9^/liter) and neutrophil fraction (81.8%, normal 40–80%). An abdomino-pelvic computed tomography (CT) with intravenous (IV) contrast revealed a distended stomach and several dilated loops of small bowel in the mid-abdomen, with multiple air-fluid levels and gradual decompression proximally and distally (Image).

CPC-EM Capsule

What do we already know about this clinical entity?

Complete small bowel obstruction (SBO) is a common surgical emergency often caused by adhesive bands that form after abdominal surgery or inflammatory disease.

What makes this presentation of disease reportable?

Our patient had no such history, and therefore the adhesive SBO likely resulted from blunt abdominal trauma sustained several decades prior.

What is the major learning point?

It is critical to obtain a thorough history of abdominal trauma from patients presenting with symptoms of obstruction yet lacking these common risk factors.

How might this improve emergency medicine practice?

Delaying surgery for complicated SBO increases its mortality; thus, recognizing unusual causes of obstruction may expedite intervention and improve patient outcomes.

There was no evidence of ischemia, but several bowel loops had inflammatory stranding and surrounding free fluid. The patient received IV isotonic fluid resuscitation and, following admission to the hospital, subsequently developed peritoneal signs. Due to concern for acute SBO with peritonitis, the patient underwent exploratory laparotomy later that day. He was found to have a fibrous band extending from the greater omentum to the jejunum, through which a 20 centimeter segment of decompressed bowel formed a closed-loop obstruction around which the jejunum was wrapped. After the entire length of the intestine was inspected and its viability was confirmed, the omental adhesion was divided and the bowel was released.

The patient’s post-operative course was complicated by mild colonic ileus and a cellulitic incisional infection that resolved following a course of cephalexin. At follow-up on post-operative day 12, he was asymptomatic and had a normal physical examination.

## DISCUSSION

Adhesions are the most common cause of SBO and thus create a substantial burden for patients and healthcare systems.^1^–^4^ Adhesions may be congenital or acquired, arising either from inflammatory conditions including appendicitis, diverticulitis, or pelvic inflammatory disease, intraperitoneal infection, or abdominal trauma.^5^ The true proportions of each etiology vary among studies, though there is consensus that a majority of adhesions are due to abdomino-pelvic surgery.^4^–^5^ Greater than 93% of patients who have undergone transperitoneal surgery develop intra-abdominal adhesions.^4^ During surgery, damage to the peritoneum and its microvasculature causes a release of serosanguinous exudate that forms a fibrinous band connecting adjacent organs or injured serous membranes.^4^ Though adhesions ordinarily disintegrate within 72 hours, injury-induced ischemia may diminish fibrinolysis and allow the band to persist.^4^

Our patient’s omental band adhesion was not likely the result of his prior pelvic surgery. The reduction and internal fixation of his pubic symphysis was entirely extraperitoneal and there was no evidence of screws transecting the peritoneum. Instead, we attribute his adhesive SBO to blunt abdominal trauma sustained during a remote MVC. Approximately 3–5% of patients treated for blunt abdominal trauma, commonly related to seatbelt use during MVCs, receive hollow viscus and mesenteric injuries.^6^–^7^ These injuries are inflicted either through deceleration and shearing of attachment points, particularly in fixed sections of the bowel like the proximal jejunum, or through compression against the vertebral column.^7^–^8^ Without uncontrolled bleeding or peritonitis, patients sustaining blunt abdominal trauma are managed conservatively, yet infrequently may develop adhesive SBO later.^7^,^9^

Adhesions that are not broken down will mature within 10–14 days.^5^ Greater than 20% of adhesive obstructions occur within one month of injury, approximately 50% develop within 1–2 years, and occasionally obstructions may occur more than 10 years after trauma.^5^,^10^ The pathophysiology for the delay of obstructions following blunt abdominal trauma is not currently understood.

Adhesive SBO often presents similarly to other acute abdominal diseases*,* with symptoms including colicky abdominal pain, nausea, vomiting, abdominal distension, and obstipation.^11^ On examination, the patient may appear dehydrated and have active, high-pitched bowel sounds, though abdominal auscultation generally has poor sensitivity and specificity for bowel obstruction.^12^ Abdominal radiographs can help to identify SBO in 50–60% of cases and is often performed as the initial imaging tool because of its relatively low expense and radiation exposure.^11^

However, CT of the abdomen is more useful for determining the location and etiology of a SBO, and therefore may be used instead of radiographs when this diagnosis is strongly suspected.^13^ Despite the inability of CT imaging to visualize most fibrous bands, it has a positive predictive value of 71% for adhesive SBO owing to the appearance of the transition zone made by the adhesion.^13^ Signs of compromised perfusion of the small bowel include tachycardia, focal abdominal tenderness, fever, and leukocytosis, though CT imaging remains the only reliable indicator of strangulation or ischemia.^11^ Because clinical presentation, physical examination, and laboratory tests cannot accurately detect complications that require rapid surgery, CT imaging is also critical for guiding treatment course.^14^

Historically, patients suspected of having SBO secondary to adhesions underwent surgery immediately due to the uncertainty of strangulation.^15^ More recently, conservative therapy with administration of IV fluids, electrolyte supplementation, and nasogastric tube decompression of the stomach has become the preferred initial management of adhesive SBO.^11^ Non-operative treatment was shown to be successful in up to 80% of cases of uncomplicated partial SBO due to adhesions.^5^ Additionally, operative interventions are associated with significant risks including enterotomy, prolonged ileus, and recurrence of adhesions resulting from iatrogenic peritoneal injury.^16^ However, although guidelines exist, there is a lack of consensus within the literature and a paucity of evidence-supported criteria dictating which patients may be safely managed conservatively.^15^ Because delay in surgery for complicated SBO increases risk of mortality, many institutions still use early laparotomy, especially in patients without a history of intra-abdominal surgery.^16^

Early identification and treatment is particularly important in the case of a closed-loop obstruction, which can quickly progress to strangulation, ischemia, and necrosis.^17^ A closed-loop SBO forms when the lumen is blocked at two contiguous points, forming a segment of intestine with no outlet proximally or distally. Abdominal distension, the most common physical examination finding in patients with SBO, is minimal with closed-loop obstructions.^1^ Furthermore, CT imaging lacks the specificity to distinguish closed loops.^13^ Many cases of closed-loop SBO therefore require exploratory surgery to make a diagnosis.

Our patient’s clinical presentation was consistent with a SBO, including symptoms of nausea, abdominal tenderness, and dehydration, yet his medical history contained no commonly identified risk factors for adhesions. We followed our institution’s standardized protocol by performing volume resuscitation and open surgery immediately after obtaining CT imaging findings consistent with obstruction and with the development of peritoneal signs consistent with bowel compromise.^16^ During the exploratory laparotomy, the patient was noted to have a single fibrous band forming a closed-loop SBO that threatened bowel strangulation, prompting lysis. Our protocol was successful in expediting surgical intervention for a case of complete bowel obstruction, abiding by current recommendations for operative management within 72 hours.^16^

## CONCLUSION

Complete adhesive SBO is a common surgical emergency that requires rapid diagnosis to minimize complications. Patients presenting with adhesive SBO may rarely have no history of abdominal surgery or inflammatory disease, and thus may pose diagnostic uncertainty. Blunt trauma can create intra-abdominal adhesions, yet when old or trivial, may not be recognized as the cause. It is therefore imperative to take a thorough history of abdominal trauma from patients who present with symptoms consistent with obstruction.

## Figures and Tables

**Image:**
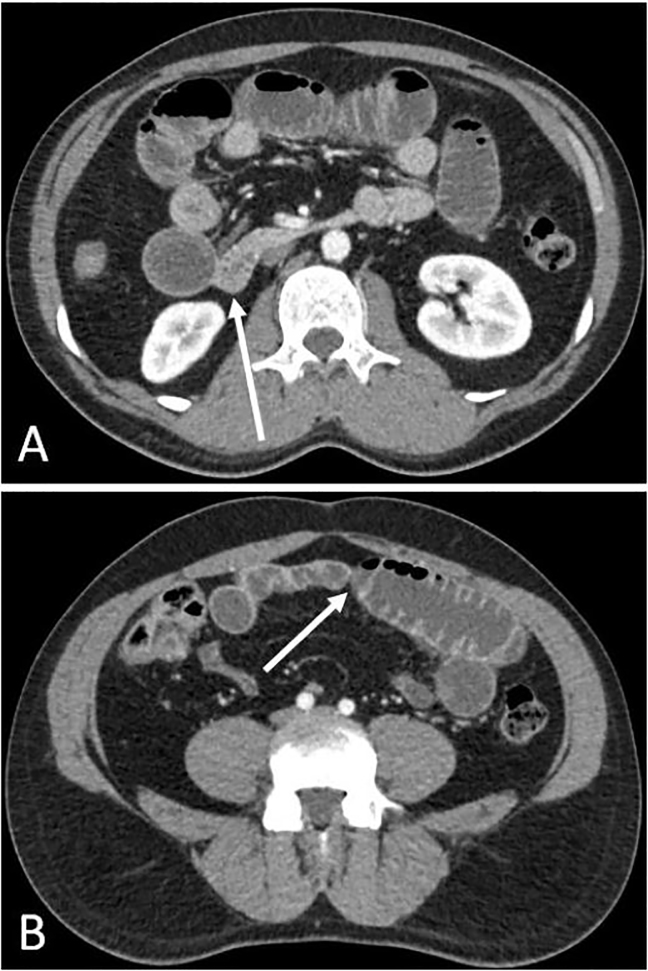
Axial view of abdominal computed tomography images showing the proximal (A) and distal (B) transition points (arrows) of a small bowel obstruction.
